# Aging as a particular case of phenoptosis, the programmed death of an organism (A response to Kirkwood and Melov “On the programmed/non-programmed nature of ageing within the life history”)

**DOI:** 10.18632/aging.100403

**Published:** 2011-12-06

**Authors:** Vladimir P. Skulachev

**Affiliations:** Belozersky Institute of Physico-Chemical Biology, Lomonosov Moscow State University, Vorobyevy Gory 1, Moscow 119991, Russia

The abstract of the paper published by T. B. L. Kirkwood and S. Melov in the September issue of *Current Biology* starts with the following categorical statement: “Compelling arguments eliminate the idea that death is generally programmed by genes for ageing” [1]. The end of the abstract is less categorical: “It is recognized that in exceptional circumstances the possibility exists for selection to favor limiting survival. In acknowledging that at least in theory, aging might occasionally be adaptive, however, the high barriers to validating actual instances of adaptive ageing are made clear” [1]. A few years ago it was hardly possible to find the latter statement in an article written by the most famous proponents of non-programmed aging. Certainly, this conclusion is accompanied by some reservations. Nevertheless, the balance between concepts of programmed and non-programmed aging seems to be really shifted to the programmed one. Such a tendency appeared in 1972 when Kerr, Willie, and Currie published their paper “Apoptosis: a basic biological phenomenon” [2]. The main message of this article was that cells of multicellular organisms are equipped with a death program. In 2002, Severin and Hyman showed that a natural signal molecule (yeast pheromone) sex-specifically kills *S. cerevisiae* cells [3]. I suggested that this phenomenon can be regarded as a precedent of programmed death of a unicellular organism [4]. Later it was found in our group that the mechanism of such death strikingly resembles apoptosis of higher organisms [5]. To explain the pheromone effect on yeast in terms of the traditional concept of non-programmed death of organisms, Kirkwood and Melov [1] assumed that yeast cells form in fact a multicellular organism. Such an explanation is hardly sufficient since (i) programmed death phenomena are now also described in bacteria [6-9] and (ii) an additional function of a pheromone as an inducer of the organism's death program was discovered in a mammal, the small marsupial *Antechinus stuartii*. Male of this rodent uses pheromone to attract females and then to kill himself after run [10]. To define all cases of programmed death of organisms, in 1997 I suggested the term "phenoptosis" [11] (for discussion, see [7, 9, 12]).

The idea that programmed death was invented by biological evolution was introduced in the end of nineteenth century by August Weismann [13], who suggested that such a death is useful for evolution as a mechanism which (i) purifies the population from weak individuals and (ii) promotes succession of generations. For sure, both these roles may be inherent in aging. However, they failed to explain why aging represents *slow* and *concerted* decline of *many* physiological functions (slow phenoptosis) rather than simple fast switching off of a single function of vital importance (acute phenoptosis).

Aesop once mentioned that a hare always runs away from a fox because for the hare this is the question of his life or death, while for the fox it is a question of a dinner. If this is the case, the fox is a not a factor of natural selection of hares. However, such a conclusion is right only for *young* hares. In 2003 I wrote: “Two young hares differing in IQ have equal chances to escape from a fox since both of them run faster than a fox. This situation is nicely described by the Russian proverb: “Syla est' – uma ne nado”. (“If you are strong, it is not necessary to be clever”). However, with age the clever hare acquires some advantage that becomes of crucial importance when the speed of running of the hares becomes slower than that of the fox. Now the clever hare has a better chance to escape and, hence, to produce leverets, than the stupid hare. This in turn will be favorable for selection of clever hares" [9].

These relationships presume that aging of muscles develops faster than that of the brain and the reproductive system. This is certainly the case for humans since here “aging atrophy of muscles (sarcopenia) begins at around 25 years” [14]. As for the immune system, its age-related decline starts even earlier (around 15 years) [15] (visual acuity, around 30 years; decrease in lung volume, around 35 years; skin elasticity, around 45 years [16]). Decline of any of these functions can, in principle, contribute to the general weakening of the human organism at ages much younger than usually considered as old. Goldsmith wrote: “Because even a relatively minute deterioration will cause a statistically significant increase in the death rate, one suspects that the evolutionary effects of aging in wild mammals begin at relatively young ages” [17]. Such a conclusion is based upon numerous observations summarized by Libertini [18-20] and Loison et al. [21] who stressed that death rates in wild mammals increase beginning at puberty.

The assumption that aging is favorable for evolvability [9, 11, 12, 17] presumes that natural selection fails to eliminate traits of the aging program that increase evolvability but are counterproductive for individuals. This is a particular case of a more general problem: how are some counterproductive traits retained in spite of pressure of natural selection? In other words: why do numerous cases of phenoptosis still exist in spite of billions of years of biological evolution? One answer might be that in any phenoptotic mechanism there is a bifunctional component which, besides its role in a counterproductive system, is required for another system of vital importance. For instance, cytochrome *c* has the *vital* function of respiratory chain electron carrier and the *lethal* function of activator of Apaf-1 involved in programmed cell death [7]. In such a case, mutations eliminating the considered component will always be lethal.

It is already clear that besides the above-described mechanism preventing elimination of counterproductive traits, there are other still unknown ways to achieve the same effect. It has been known for a long time that the death of soybean plants occurring soon after maturation of seeds can be prevented by depodding [22] or deseeding [23]. In both cases the lifespan of the plant is greatly increased. An interesting experiment was carried out by Nooden and Murray [24]. When a soybean plant was depodded except for a single pod cluster in the center of the plant, the pod cluster induced yellowing of the nearest leaf only, whereas the rest of the plant remained green. The effect remained even if the petiole contained a zone treated with a jet of steam killing the phloem. This treatment inactivated transport of compounds from leaf to pods occurring via phloem (a living tissue) but not from pods to leaf, which occurs via xylem (an already dead tissue at functional maturity of plants). This indicates that pods induce senescence by producing a dead signal or a poison killing leaves. This observation is in obvious contrast with a statement of Kirkwood and Melov: “There is a little evidence that semelparous (capable of only single reproduction) organisms are *actively* destroyed once reproduction is complete” [1].

Senescence of soybeans is a fast process that takes about ten days. The lifespan of this plant is about 90 days, which is greatly increased by depodding (deseeding). A similar increase was revealed in many other semelparous plants including *Arabidopsis thaliana*, the classical model species for plant physiologists and geneticists. Just this organism was recently used by Melzer and coworkers from the Department of Plant System Biology of Ghent University [25], who disprove one more point in Kirkwood and Melov's reasoning against programmed aging: “Yet among the many gene mutations that have been discovered that affect lifespan, often increasing it significantly, none has yet been found that abolishes aging altogether” [1]. The Belgian authors reported in Nature Genetics, 2008, that a plant having mutations in the *soc1* and *full* genes simultaneously switches from sexual to vegetative reproduction, does not form seeds, and does not die due to seed-induced senescence. The lifespan increases from 80-90 days to practically infinity as in perennial trees or bushes reproducing with rhizomes. In fact, the *soc1 full* mutant forms woody stems and rootstocks, it becomes much larger than the wild type *A. thaliana*, changing from a grass with a single basal rosette of small leaves to a highly branched shrub with many aerial rosettes formed by rather large and thick leaves. Inflorescence meristems are reversed to vegetative meristems. Secondary growth appears, being mediated by cambial activity absent from the wild type. The authors speculate that originally the species *A. thaliana* appeared as a perennial shrub vegetatively reproducing with rootstocks and competing with other shrubs and trees. The modern version of the species became a small short-lived grass reproducing with very light seeds transmitted with wind to open soil. If an opening is fresh, the seeds quickly germinate to give tiny plants growing with no competition with other plant species, which are still absent from the opening. The grass-type *A. thaliana* is short-lived, being killed by its own seeds. Such death might be necessary to accelerate succession of new generations and, hence, their evolution. Apparently, the modern form of *A. thaliana* appeared quite recently, since the program for its preceding (immortal) form is still conserved in the genome of this plant. Another advantage of short lifespan of the modern *A. thaliana* might be that it guarantees for this organism a life under comfortable conditions when competition with other species for the niche is excluded. Small *Arabidopsis* can hardly complete with other grasses and, if long-lived, will inevitably fail in the struggle for existence as if the fox will always kill not only stupid but also clever hare in our hares versus fox case.

In any case, the study of *A. thaliana* mutants clearly shows that senescence and death of a higher organism can be cancelled by means of inactivation of a few genes in its genome^1^.

The *A. thaliana* case is hardly an exception. Melzer et al. [25] mentioned that “in angiosperms, the perennial woody habit is believed to be the ancestral condition from which annual herbaceous lineages have evolved several times independently [26]. Conversely, evolution from annual herbaceous ancestors to perennial woody taxa has also repeatedly occurred. For example, in various annual herbaceous lineages, such as *Sonchus* and *Echium*, woody perennial species evolved on isolated islands from their continental annual ancestors [27-29]”. Among perennial plants there are examples of organisms vegetatively reproducing for many years, then switching over to the sexual reproduction and dying when their seeds mature. Several species of bamboo have fixed lifespans determined by the time of inflorescence [13]. This time is species-specific. It varies from 6 to 120 years in species that belong to a single genus. A similar situation was described for some other perennial plants, e.g., *Agave* and the Madagascar palm *Ravenala madagascariensis*, flourishing in the 10^th^ and 100^th^ year of life, respectively, and dying after maturation of seeds.

Certainly, one might consider these observations as plant-specific exceptions, assuming that phenoptoses are specific to plants only^2^. However, numerous cases of acute phenoptosis are reported also for the animal kingdom [7, 9-12, 16, 30].

The great majority of reservations formulated by Kirkwood and Melov [1] can be applied when acute phenoptoses are considered. If they are groundless, it is not clear why they should be accepted when aging is considered. However, one argument should be discussed especially because it really may be specific for aging as slow phenoptosis. I mean “huge interindividual differences” in ages of death [1]. On the face of it, the standard deviation of lifespan of humans is much larger than, say, the age when menarche occurs in girls (an example of an event programmed by an ontogenetic mechanism). However, simple logic indicates that in such a calculation one must normalize the deviation value by the age. We compared absolute values of deviations of ages when menarche and menopause occurring in a particular person and found that they are much larger for menopause. However, the difference disappeared when the absolute value of the age (years) was taken into account. The deviation of lifespan, normalized in the same manner, showed that it is slightly higher than those for menarche and menopause, but this is hardly surprising since, besides age-dependent (programmed) death, age-independent death cases contribute to factors determining lifespan. In any case, normalized deviation in ages of death is of the same order of magnitude as that of ages of menarche and menopause [31].

The Conclusions section of Kirkwood and Melov's paper starts with the following statement that I completely agree with: “There is, it must be acknowledged, an instinctive attraction to the idea that aging is programmed. Aging is widespread across species and applies universally to all individuals within a species in which it is observed. There is also reproducibility about changes that occur with aging…” [1]. I may only add that, if aging is programmed, it can be retarded, prevented, and perhaps even reversed by treatments interrupting execution of this program, just as we already can interrupt programs of cell death. In other words, programmed aging can be cured like a disease. As for the concept of non-programmed aging, assuming occasional accumulation of stochastic injuries as its reason, it is quite pessimistic for finding any way of successful treatment. Here we simply observe and describe such a process without the possibility of improving the situation. This is why we must analyze all the possibilities of the programmed aging concept rather than treat it as an idea that can at present be eliminated due to existence of “compelling arguments”. It seems to me such arguments are still illusive rather than compelling.

## Footnotes

1 Remarkably, the very fact that the death of semelparous plants is caused by their seeds literally confirms a famous maxim of Weismann that highly organized living organisms contain “seeds of death” [13].

2 In the 1930s, when DNA was found in animal and RNA in plants, the former and the latter were considered in biochemical textbooks as polymers specific for each of these kingdoms of living organisms.
